# Dr. Frank E. Hill

**Published:** 1915-03

**Authors:** 


**Dr. Frank E. Hill,** Western Reserve 1883, died at his home in Buffalo, Feb. 3. He was born in Blairsville, Pa., May 5, 1862. After his graduation, he took a post-graduate course at the Bellevue Hospital and thence came to Buffalo where he has resided ever since. He was a member of the German Hospital staff and of the Medical Society of the County of Erie and was a Mason of high standing.

